# How social media exposure constructs social confidence: An empirical study on impact, mechanisms, and multilateral relationships

**DOI:** 10.1371/journal.pone.0308745

**Published:** 2024-09-17

**Authors:** Yani Liu, Kang Hu

**Affiliations:** 1 Department of Journalism, School of Humanities and Arts, Southwestern University of Finance and Economics, Chengdu, China; 2 Research Institute of Social Development, Southwestern University of Finance and Economics, Chengdu, China; Zhejiang University, CHINA

## Abstract

Social confidence functions as a vital spiritual force in fostering the positive and healthy evolution of society. This paper explores how social media exposure contributes to the construction of social confidence within the framework of Media-system Dependency Theory. The research unveils the following key findings: (1) Social media exposure positively facilitates social confidence; (2) Group efficacy and group cohesion, perceived as manifestations of cognitive divergence between "efficacy" and "collective" within collective efficacy, both serve as mediating mechanisms influencing the impact of social media exposure on social confidence. The Channel Testing of Causality confirms that group cohesion plays a significantly more crucial role as a pathway compared to group efficacy; (3) The differentiated impact of social media exposure, encompassing Tencent WeChat and Sina Weibo, materializes in distinct components of social confidence and follows different influence pathways.

## Introduction

A robust social confidence holds significant implications for the health, stability, and sustained development of a society [1]. In the present era, China’s development is marked by the coexistence of strategic opportunities and concurrent challenges, coupled with a surge in unpredictable and unforeseeable factors. The intricate and ever-changing social environment has contributed to the emergence of societal psychological states such as confusion, anxiety, and depression among the public. To adapt to the constantly evolving new social transition phase and address emerging social risks, social members need to strengthen and boost social confidence. This not only provides the public with a sense of calmness, security, and control, maintaining a positive and upward psychological health status [2] but also fosters identification with the institutional advantages in the country, enhancing the intention to support, adhere to, and uphold the party and state policies [3].

Governance of social confidence requires leveraging the propagative and guiding role of new media. People are unable to directly experience various aspects of the real environment and rely on the pseudo-environment constructed by new media to perceive society and form an overall social attitude [4]. While the media disseminates information about the societal status and changes, maintaining the public’s overall confidence in the social system is its crucial responsibility [5]. Social media, as the mainstream platform of new media, with its characteristic of "everyone as a medium," not only influences social relationships but also shapes the formation of mimicry environments at various levels [6]. It holds significant value in constructing social confidence.

Research on social confidence has received widespread attention and active exploration in the academic community. Existing studies primarily unfold in four aspects: firstly, there are descriptions and comparative analyses of the current status of social confidence for specific groups or geographical areas [7]. Secondly, endeavors have been undertaken to articulate the conceptual underpinnings of social confidence, establishing mature measurement dimensions and indicators [8]. However, empirical research frequently exhibits a tendency to gauge it through partial dimensions, overlooking its holistic facets. This is apparent in the emphasis on real-life situations during measurement, lacking comprehensive investigations into diverse facets of societal development, and notably neglecting inquiries into the public’s future psychological expectations. Thirdly, there is a concentrated exploration of the potential antecedent variables of social confidence, particularly focusing on individual life experiences [9], government performance [10], and other social reality factors. The relationship between the media and social confidence has not received sufficient attention. Fourthly, in the limited exploration by a few scholars into the relationship between mass media and social confidence, the mechanism of the role of perceived media trust has been verified [11]. However, research validating the effectiveness of other potential mechanism variables is relatively scarce. Upon reviewing the aforementioned related studies, the following shortcomings are identified: an emphasis on real environments rather than mimicry ones, overlooking the constructive role of mass media in shaping social confidence, failure to explore diverse causal pathways between the two, and a lack of systematic measurement in assessing social confidence, thereby limiting the reliability and replicability of existing research findings.

To address the aforementioned research gaps, this paper aims to investigate the impact of social media exposure on social confidence guided by the Media-system Dependency Theory and seeks to validate the explanatory power of collective efficacy in the logic of social confidence formation. Firstly, this paper comprehensively expounds on social confidence from the perspectives of time and social events, utilizing a systematic measurement, thus compensating for past research’s neglect of the temporal characteristics and diverse components of social confidence. This facilitates a correct and comprehensive understanding of the conceptual connotations of social confidence. Secondly, by exploring the impact of heterogeneous social media exposure on social confidence, this paper provides robust evidence to affirm and strengthen the positive predictive role of social media exposure in social confidence. Furthermore, by investigating the mediating role of collective efficacy in constructing social confidence, the paper, while fully recognizing its conceptual divergences including group efficacy and group cohesion, employs mediation testing and Channel Testing of Causality to validate and compare their mechanisms with other potential pathways, thus guiding the identification of critical construction pathways. Additionally, the paper discovers in its exploration of the relationship between heterogeneous social media exposure, including Tencent WeChat and Sina Weibo, and social confidence that the multilateral relationship lies not in the traditional understanding of main effect influences, but in different components of social confidence and influence pathways, thereby extending and deepening the understanding of this multilateral relationship.

## Literature review and research hypotheses

### Social confidence

To explore the constructive role of social media in shaping social confidence, it is important to have a correct understanding of the conceptual connotations and characteristics of social confidence. Social confidence refers to a psychological force that enables citizens to believe in the future realization of a certain entity or goal, primarily involving the public’s recognition, trust, psychological state, and the stable psychological expectations formed towards a specific agent, object, or entity [12]. A systematic examination of existing research reveals that many studies use time or social events as entry points to understand social confidence. From a temporal perspective, social confidence is intricately linked to the societal development status and the process of social change [9]. The Theory of Perception of Social Change suggests that people’s perceptions of social change include not only an understanding of the current development but also a folk comprehension of developmental patterns and predictions about the future direction of societal development [13]. Thus, judgment of the current societal conditions and beliefs about future development constitute essential elements of social confidence. However, some studies, while considering the premise of the "future based on reality," overly emphasize the realistic aspects of social confidence, leading to conceptual confusion. For instance, equating social confidence with social trust [14]. It is noteworthy that temporality is a crucial feature distinguishing social confidence from other social emotions. As a future-oriented emotion, social confidence not only manifests as the influence of present reality on future predictions but also as the feedback effect of the anticipated future on present reality through a sense of certainty [15]. Therefore, this paper contends that a comprehensive understanding of both the reality and future dimensions of social confidence is unavoidable.

From the perspective of social events, based on the different referents of confidence according to the attitude subject, event-based social confidence primarily includes national-event confidence, societal-event confidence, and personal-event confidence [16]. These three dimensions, integrated and complementary to each other, are crucial reflections of people’s overall attitudes and opinions toward society. In both qualitative and quantitative research, scholars’ exploration of social confidence under the logic of events is not yet mature. Some researchers use a single dimension or a few key indicators to represent social confidence. Relevant studies mainly revolve around topics such as economic confidence [17] and party-government confidence [18]. What’s more, some scholars do not directly focus on social confidence but instead choose proxy variables like life satisfaction for related discussions [1]. To avoid a one-sided understanding of social confidence, it is necessary to comprehensively understand social confidence in various events and draw on scientifically validated measurement methods from existing mature scales.

Therefore, considering the previous research’s tendency to emphasize solely the present dimensions of social confidence or partial indicators of events, this paper aims to advance the understanding of social confidence in terms of its temporal characteristics and diverse components of events. By incorporating both temporal and social event perspectives, the study comprehensively examines the role of social media in constructing social confidence. Specifically, this paper, considering social confidence under the logic of time as a comprehensive evaluation of various social events during a specific period, we prioritize the understanding of social confidence from a temporal perspective. In robustness tests, we use the event-based perspective for measurement to verify and strengthen the constructive role of social media exposure on social confidence.

### Social media exposure and social confidence

Media-system Dependency Theory posits that the foundation of media influence lies in the relationships among the social system, media system, and audience system. The dynamism and complexity of the social environment, along with the resulting uncertainty, lead individuals to rely on the informational resources provided by the media to comprehend society. This dependency results in individuals’ social cognition and attitudes being significantly influenced by the media system [19]. Social media, defined as a series of network applications built on Web2.0 technology and ideology, enables users to create and communicate user-generated content [17], thus representing a pivotal aspect of this framework. It highlights two essential elements: social presence and self-presentation [20], while also offering opportunities for social interaction, learning, and community co-creation [21]. As a highly popular and engaging form of new media, social media plays a crucial role in shaping social confidence. On the one hand, the convenience and timeliness of social media enable people to access richer news information, allowing the public to form evaluations and expectations of society while understanding its dynamics and public sentiment. On the other hand, considering the embedded network relationships within social media and the networked connections formed under individual goals such as socializing, obtaining information, learning, and collaborating, individuals’ social attitudes are influenced both by the members of the interactive network and by the assimilation effects of the attitudes within their affiliated groups.

Social media’s functional advantage in enhancing social confidence lies in its greater potential to connect heterogeneous subjects and broaden the informational scope of audiences [22]. Regarding the official opinion field, some scholars argue that the foundational task of constructing social confidence involves clarifying facts and emphasizing the need to address the issue of information asymmetry between the government and the public. By leveraging social media, transparency in the operation of government public affairs can be maintained, ensuring the public’s right to information, enhancing public trust, preventing subjective judgments on societal issues, and suppressing the spread of false information and negative social sentiments [12]. For the public opinion field, individuals engage in opinion exchange and societal discourse on social media platforms. Through this, they discover and receive support in terms of information, emotions, experiences, and stances. This not only helps individuals resolve confusion and eliminate uncertainty but also raises public awareness about the presence of social groups capable of collectively resisting societal risks. Consequently, this enhances their sense of control over the social environment and confidence in predicting societal developments [17].

However, some scholars, considering the media environment and its informational characteristics, make competitive judgments contrary to the positive relationship between social media and social confidence mentioned earlier. According to the hypothesis of the "mean world syndrome," the more frequently people are exposed to the mass media, the more they are influenced by its negative information, leading them to perceive the world as violent and dangerous than it actually is [23]. The media, an important social amplification station of risk, also intensifies people’s awareness of societal risks, resulting in a misjudgment of the overall reality of society. In the context of social media, fragmented social relationships can easily trigger the emergence of disharmonious voices in the network, affecting the stability of public space [24], exerting inhibitory effects on the factors contributing to the formation of positive social confidence. Also, the distinct "selective disclosure" feature of social media tends to induce "group polarization" in public opinions [11]. When extreme, irrational, responsibility-weakened, or purposefully polarized opinions continue to ferment or are manipulated by malicious actors, negative social effects arise, subsequently leading to the reverse development of social confidence [25].

In light of the aforementioned, there is currently a divergence of opinions in academia regarding the relationship between social media exposure and social confidence. Considering the specific context of China, this paper tends to infer a promoting role of social media in shaping social confidence. This inclination arises mainly from the collectivist cultural background in China, where people emphasize relationship-based interdependent selves, and the abundant relational information provided by social media becomes a crucial basis for individuals to define themselves, understand others, and even predict societal trends [26]. In addition to its economic and public attributes, the media in China carries significant political attributes. Social media, as a highly pervasive and multifaceted information dissemination medium embedded in the societal system, also assumes various governmental functions [27]. This political nature requires the media to adhere to the correct public opinion guidance, follow positive propaganda policies, and play the roles of "gatekeepers" and "social safety valves." This is not only conducive to alleviating and managing societal emotions, maintaining social harmony and stability but also contributes to propagating the essence of a socially healthy and uplifting nature, actively boosting social confidence [28]. In conclusion, the following hypothesis is proposed:

H1: Social media exposure positively influences social confidence.

Building upon the affirmation of the positive predictive role of social media exposure on social confidence, this study proceeds to examine the heterogeneous media effects on social confidence. According to financial report data up to the fourth quarter of 2023, Tencent WeChat boasts a monthly active user count of 1.343 billion, while Sina Weibo has reached 598 million, establishing them as China’s largest and most extensively utilized social media platforms. The primary distinction between WeChat and Weibo lies in their connections within their respective social networks. According to the Strength of Weak Ties, WeChat operates as a strong-tie social network, while Weibo operates as a weak-tie social network [29]. Specifically, WeChat employs a two-way authentication mechanism, requiring mutual consent between users for connection establishment, primarily consisting of friends, relatives, and coworkers with direct and high interaction in real-life, also known as strong-tie communities [30, 31]. In contrast, Weibo utilizes a one-way authentication mechanism, where relationship establishment does not necessitate the permission of the followed user. Interpersonal relationships on Weibo are typically based on common interests or topics, resulting in a more diverse network of connections, also known as weak-tie communities [30, 31]. This indicates that compared to WeChat’s personalized and exclusive nature, Weibo’s weak ties offer the public a wider range of information resources and heterogeneous viewpoints [32].

Some scholars investigating WeChat’s impact on contentious politics have found that discussions often restricted to non-challenging political topics, driven by concerns for reputation protection, information opacity, and interpersonal monitoring [33]. Similarly, privacy considerations vary across social media platforms. Due to the presence of strong tie audiences on WeChat, individuals tend to idealize themselves and project positive images, limiting their speech tendencies and expressing more neutral and conservative opinions on social events [34]. Conversely, on Weibo, the public with weak ties mitigates concerns about accountability for their speech, leading to more pronounced and radical public opinions. Therefore, whether in terms of topic restrictions or opinion tendencies, WeChat exposure is more likely to positively influence social confidence compared to Weibo exposure. Despite the diverse information and sometimes controversial public opinions found on Weibo, which may lead to divergent social attitudes and potentially undermine social confidence [34], exposure to varied viewpoints and perspectives also promotes rational discourse and a comprehensive understanding of societal issues. This, in turn, fosters engagement in public affairs, igniting social trust and a sense of civic responsibility, thus bolstering civic culture and systemic support and ultimately strengthening social confidence [35].

In addition to individual ordinary netizens mentioned earlier, both WeChat and Weibo also host a large number of self-media and official accounts, which indicate that social media serve not only for interpersonal communication but also for information dissemination and amplification of appeals. Self-media facilitates timely information transmission, collision of diverse thoughts, and accessing social support [36], but more scholars still argue that it hinders the construction of positive social confidence. Self-media has characteristics such as low entry barriers, uneven staff literacy, simplified information release, and lack of rigorous scrutiny [37]. In such a free and complex media environment, there is a risk of public opinions being temporarily concealed. As emotionally charged or provocative content continues to ferment, its impactfulness and uncontrollability directly affect public emotions and social order [38, 39]. Furthermore, some self-media intentionally fabricate, exaggerate, or distort information for traffic and commercial interests, leading to online rumors that can cause public panic and social unrest in the absence of official information [40].

However, compared to self-media accounts and other unofficial accounts verified through platform authentication mechanisms, official accounts enjoy higher credibility, which is pivotal for their ability to dominate positive public sentiment [11, 41]. Official accounts such as government affairs and news media wield greater authority, enabling them to disseminate verified, accurate, and comprehensive information, thereby correcting misinformation and calming public expectations to promote social stability [40]. Some scholars also indicate that exposure to official information enhances public political efficacy, political trust, and subjective well-being [22, 42]. Importantly, their transparency enhances government credibility, which in turn boosts media credibility, fostering a virtuous cycle that shapes positive social confidence [41]. In the context of China, official accounts serve as new forms of traditional media, presenting content that aligns with their role as the mouthpieces of the party and government. New media platforms like WeChat and Weibo similarly possess political attributes, serving the interests of power and political systems. Due to their lack of legitimate interviewing rights and regulation by relevant departments, they function solely as tools for aggregating and disseminating official information [43]. Therefore, despite not being the primary agenda setters, the party and government still manage to dominate public opinion trends through their authority, thus ensuring to some extent the positive development of social mentality [44].

In summary, after discussing the complex user composition, this paper concludes that although some self-media generate false information and uncontrollable public opinion, WeChat and Weibo generally exhibit a positive trend in public sentiment. This is due to ordinary netizens’ positive self-presentation and perception of efficacy, guidance from official media, and platform regulation, which collectively enhance social confidence. Therefore, the following hypotheses are proposed:

H2a: WeChat exposure positively influences social confidence.
H2b: Weibo exposure positively influences social confidence.

### Mechanisms: The mediating role of collective efficacy

Research indicates that social confidence is the outcome of mutual influence among individuals, and the complex network relationships between people form the structural basis of social confidence [16]. The accurate interpretation of the formation logic of social confidence emphasizes understanding the impact pathway of social media exposure on social confidence from the perspective of "individuals within the group." Collective efficacy, a collective sense of power with the potential to shape and transform society, involves group members believing in their ability to alter the situation and destiny of their society, in turn fostering a relatively positive social psychological state.

Tracing the evolutionary process of research on collective efficacy reveals a shift in theoretical exploration within the academic community—from a simple emphasis on the intensity of network relationships to a focus on goal-oriented community mobilization capacity. Two main perspectives have emerged: the first perspective centers on "efficacy," aligning with the mainstream view among scholars based on social cognitive theory. It refers to a group or individual within a group’s perception of their ability to carry out specific actions to achieve expected social outcomes [45, 46]. The second perspective emphasizes the "collective" aspect, highlighting the social relationships and structures in which group cognition occurs. It equates collective efficacy with the combination of social cohesion and informal social control orientation, emphasizing mutual attraction among group members and collective commitment to common goals [47]. In reality, these two perspectives manifest as different emergent states—either focusing on tasks or relationships within the group. It is essential to independently investigate them as distinct factors [48]. To clarify the referent object as the group in social media rather than the nation or culture, this paper distinguishes collective efficacy into group efficacy and group cohesion, and subsequently explores the mediation roles of both in the relationship between social media exposure and social confidence.

Regarding group efficacy, it plays a significant role in shaping a group’s initiative, effort, and the duration of effort when engaging in specific tasks or contexts [49]. Social media provides opportunities for the public to engage in continuous, open, and visible discussions on public affairs, fostering group efficacy through positive information exchange and collaborative dialogues [50]. For citizens with high group efficacy, frequent social interactions offer them more opportunities to understand, coordinate, and share the diverse abilities and resources possessed by group members. By mobilizing various resources to overcome common challenges [51], individuals with high group efficacy exhibit better receptivity and resilience when facing larger challenges. Consequently, they tend to have relatively optimistic cognitive judgments regarding the completion and performance of group tasks [52]. Simultaneously, high group efficacy increases people’s involvement and influence in social issues, fostering a supportive attitude toward government public policies and social governance actions aimed at problem-solving [53]. It also contributes to advancing the realization of national policy goals in the process of triggering informal political participation behaviors among citizens [49]. Therefore, group efficacy endows individuals with a positive perception of their ability to adapt to and change society, facilitating the establishment of positive social confidence.

Regarding group cohesion, it implies the united trust and embedded group norms among its members. Leveraging the dense social networks and extensive reciprocal exchanges facilitated by social media, individuals come together for common purposes or actions, forming subjective norms and collective commitments through internalization processes [47]. The democratization of information on social media also provides a potential space for information dissemination and agenda-setting, contributing to the advancement of collective awareness regarding specific social public affairs [54]. Groups with high cohesion are relatively stable, and individuals affiliated with such groups experience a greater sense of security and less anxiety. Empirical studies have shown that the key to enhancing social confidence in public safety events depends more on individuals’ judgments of social cohesion and moral consensus than on reducing their safety concerns [55]. Moreover, some scholars argue that cohesive social relationships strengthen people’s identification and perceived legitimacy of the national political system, as well as enhance confidence in the current operation of society [56]. Therefore, the social endorsement and consensus brought about by group cohesion contribute to maintaining and building public social confidence. In summary, the following hypotheses are proposed:

H3a: Group efficacy plays a mediating role between social media exposure and social confidence.
H3b: Group cohesion plays a mediating role between social media exposure and social confidence.

## Methodology

### Data source

This study employed a questionnaire survey on the Credamo data platform to collect research data. This platform, affiliated with the Market Research Branch of the China Information Industry Association (CMRA), holds licenses and registration from the Beijing Internet Content Provider (ICP). It complies with industry norms and ethical standards, featuring well-defined user privacy protection policies and stringent data sharing mechanisms. With users across all provinces and regions of China, the platform ensures both reliable data quality and extensive data resources. To ensure respondents’ informed consent, participants were required to click the "Confirm" button after reading the questionnaire introduction, indicating their agreement to participate in the survey. During the pre-survey phase, 50 questionnaires were initially distributed on February 15, 2023, and adjustments were made to questions with ambiguous or inappropriate wording based on feedback from the surveyed individuals and the results of reliability tests. In the formal survey stage, starting from February 17, 2023, until the end of the month, a total of 4 survey rounds were conducted on the Credamo data platform, accumulating 1,267 survey questionnaires (see S1 File). Two parts of invalid samples were screened and removed: a single-choice screening question (Q21) was set in the questionnaire, "This question is to check if you are answering seriously, please choose ’dissatisfied,‴ and 11 samples that did not meet the response requirements were automatically rejected through system screening. Subsequently, samples displaying signs of non-serious or irrational responses, such as daily media exposure exceeding 24 hours, online exposure time less than social media exposure time, and contradictions in positively and negatively coded questions, were manually screened, resulting in the rejection of an additional 81 samples. After removing invalid questionnaires, a total of 1,175 valid questionnaires were obtained, yielding an effective sample rate of 92.74%. Given the dispersed and complex composition of social media users, absolute random probability sampling presents significant challenges. This study followed the convenient sampling method commonly used by previous scholars on online data platforms, while striving to ensure sample heterogeneity. Sampling results demonstrate the sample’s characteristics of youthfulness and diversified demographic features, such as gender and education, indicating its representativeness overall.

### Variable descriptions

#### Dependent variable: Social confidence

Drawing on Keller et al.’s study [3], social confidence was assessed through two dimensions: present confidence and future confidence. The former comprised four items: "Our society is capable of addressing future social issues," "The future safety and security of our people are guaranteed," "We live in a secure and reliable era," and the reverse-coded item "Current affairs seem to be increasingly out of control" (*α* = 0.775). The latter consisted of two items: "The future society will be functioning normally as well as today" and "Our society has a bright future" (*α* = 0.715). Respondents indicated their degree of agreement with these statements on a scale from "completely disagree" to "completely agree," with values assigned from 1 to 6. The measurement of social confidence was derived by averaging the scores of these six items. The average of the first four items represents present confidence, while the average of the last two items indicates future confidence. Subsequent sections utilized mean scores similarly for the measurement of multi-item variables.

#### Independent variable: Social media exposure

Following the approach outlined by Lu and Zhu for measuring media usage [57], respondents indicated the frequency of their "social media usage" with values assigned from 1 to 5 corresponding to "never use," "several times a year," "several times a month," "several times a week," and "several times a day," respectively. To capture heterogeneous social media exposure, WeChat and Weibo exposure were separately quantified. Drawing inspiration from the option design of Question a28 in the 2017 China General Social Survey (CGSS)—China’s earliest nationwide, comprehensive, and continuous academic survey project—responses to the question were coded from 1 to 5, reflecting the spectrum from "never" to "very frequently."

#### Mediating variables: Group efficacy and group cohesion

The measurement of group efficacy drew inspiration from studies by Halpern [50] and Lee [58]. Questions related to intrinsic group efficacy included "Collective actions have a significant impact on public affairs" and "Collective actions by people can improve society." External group efficacy was assessed through questions such as "If enough people demand change, the government and relevant departments will respond to those demands" and "If enough people demand change, the government and relevant departments will take measures." As for the measurement of group cohesion, modifications were made to the items based on the studies by Browning et al. [47] and Lee et al. [59]. These modifications were necessary because previous measurements defined groups as fixed and bounded entities, where individuals within the group shared the same geographical location or objectives. Whereas, online media exposure tends to foster a boundary-less sense of group cohesion. Hence, the adjusted questions included "I am willing to help others," "I have close relationships with people around me," "I get along harmoniously with people around me," "I believe others can be trusted," and "I am willing to stay in touch with people around me in the future" (*α* = 0.705). Questions regarding group norms included "There should be clear behavioral norms in the group" and "Members in the group should be clear about their responsibilities, and behave orderly." Response options for the above questions ranged from "completely disagree" to "completely agree" and were coded sequentially from 1 to 5.

#### Control variables

Considering the impact of individual characteristics on social confidence, this study controlled for variables such as gender, age, ethnicity, education level, income, residence, political affiliation, and subjective social status. The coding details were as follows: Regarding gender, "female" was coded as 0, and "male" was coded as 1. For age, the original data provided by respondents were used. Ethnicity was coded as "minority" (0) and "Han Chinese" (1). Education level was coded as "below college" (0), "bachelor’s degree" (1), and "master’s degree or above" (2). Monthly income was coded based on ranges: "0–3000 yuan" (1), "3001–5000 yuan" (2), "5001–8000 yuan" (3), "8001–10000 yuan" (4), "10001–15000 yuan" (5), and "above 15001 yuan" (6). Residence was coded as "counties, towns outside the urban area of the county or city, and rural areas" (0), "prefecture-level and county-level urban area" (1), "provincial capital urban area" (2), and "direct-administered municipality urban area" (3). Political affiliation was coded as "Non-Communist Party of China (CPC) member" (0) and "CPC member" (1). Subjective social status was coded from "lowest" to "highest," following the questioning and options in Question a43 (2017 CGSS), with values assigned from 1 to 10. The study addressed sample distribution imbalances (including education level, residence, and income) by merging certain options, and dummy variable treatment was applied during empirical analysis for the former two.

#### Potential channel variables

In order to fortify the mediating effects within the causal relationships, Channel Testing of Causality was employed during the robustness check. This "seemingly mediating effect test" involved examining the explanatory power of potential channels on the main effect model, providing tentative evidence for the mediating role of collective efficacy. Considering the explorations into the intrinsic mechanisms of social confidence by scholars such as Liu [1], Zhang et al. [11], Bi and Chu [18], media trust, social capital, and sense of social fairness were selected as potential channel variables. The specific coding methods were as follows: For media trust, based on the quantification method proposed by Su [60], respondents were asked about their level of trust in official news media, scored using a five-point Likert scale (1 = strongly disagree, 5 = strongly agree). For social capital, following the measurement in Question c46 (2017 CGSS) by Wang and Zhou [61], respondents were asked about the number of contacts they make through the internet on working days. Options such as "none," "0–4," "5–9," "10–19," "20–49," and "50 or more people" were assigned values from 1 to 6. For the sense of social fairness, based on the measurement question and option design in Question a35 (2017 CGSS) by Su [62], respondents were asked to evaluate the overall fairness of society (1 = completely unfair, 5 = completely fair). Samples choosing "do not know" (n = 4) were treated by removing them as outliers.

### Basic model specification

The choice of the multivariate linear regression model was motivated by two main factors. Firstly, the study’s dependent variable was social confidence, while the independent variables included social media exposure and multiple demographic control variables. Secondly, considering the cross-sectional nature of the data, this model was selected for its ability to effectively manage the complex relationships between multiple independent variables and the dependent variable in a static data setting, demonstrating its suitability for analysis.

The model specification is as follows:

$$Confidence={\beta_{0}+\beta }_{1}SocialMedia+\gamma CONTROLs+\varepsilon$$ (1)

Where *Confidence* represents a 3×1 matrix denoting social confidence, with matrix elements including overall, present, and future confidence. *SocialMedia* signifies social media exposure, while *CONTROLs* denote control variables, including gender, age, ethnicity, education, income, residence, political affiliation, and subjective social status, where education and residence are dummy variables. *ε* denotes the error term. The basic model of this study mainly focuses on the significance of *β*_*1*_.

### Analysis strategy

This study utilized Stata 15 software as the statistical analysis tool. The main analytical methods employed included descriptive statistical analysis and multivariate linear regression analysis. The empirical testing process of the research hypotheses mentioned above comprised three parts: main effect analysis, mediation analysis, and heterogeneity analysis. The first part examined the predictive effect of social media exposure on social confidence. Robustness tests such as variable substitution and propensity score matching were conducted. For the second part, stepwise regression and bootstrap mediation analysis were initially employed to assess the explanatory power of group efficacy and group cohesion as mechanisms for social confidence. Subsequently, Channel Testing of Causality was applied to address potential estimation biases in mediation analysis based on linear regression. The third part examined the direct impact and influence pathways of heterogeneous social media exposure on social confidence.

### Ethical considerations

Our observational study utilized a questionnaire survey method, with no manipulation of respondents’ experiences or accounts. Ethical principles outlined in the Declaration of Helsinki were followed: Firstly, respondents were fully informed of the survey’s theme and purpose to ensure informed consent. Anonymous questionnaires were used to maintain confidentiality, and results were strictly used for research purposes without disclosing personal information, safeguarding respondents’ privacy rights. Participation was voluntary, with the option to withdraw at any time. Secondly, questionnaire content was derived from established scales, avoiding subjective composition. Thirdly, all survey questions were reviewed by the Credamo data platform to ensure compliance with ethical standards, with potential violations leading to rejection of distribution requests.

## Empirical testing and results analysis

### Descriptive statistical analysis

**Fig 1:**
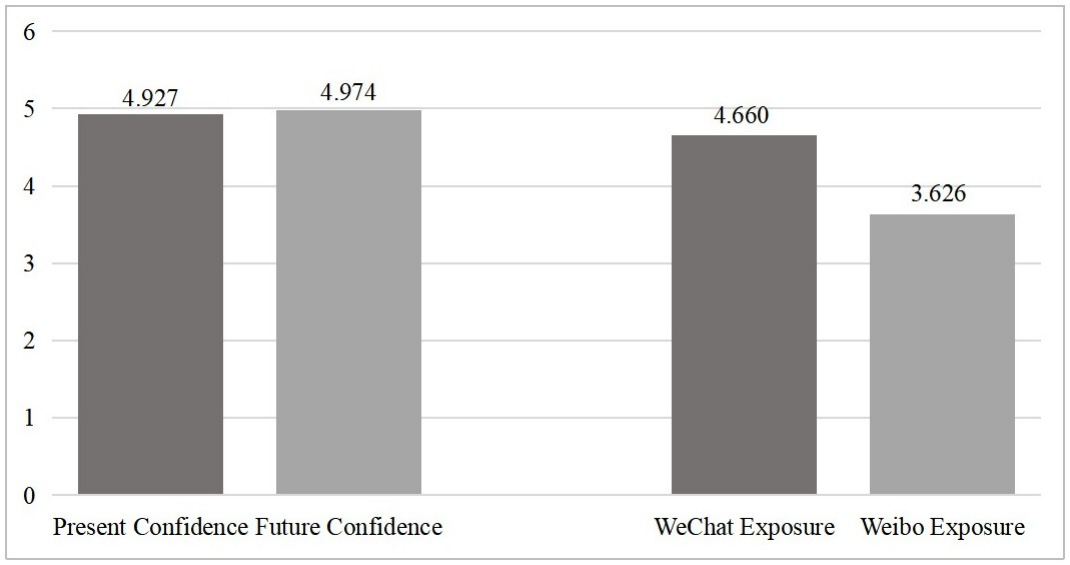
T-test analysis of independent and dependent variables.

**Fig 2:**
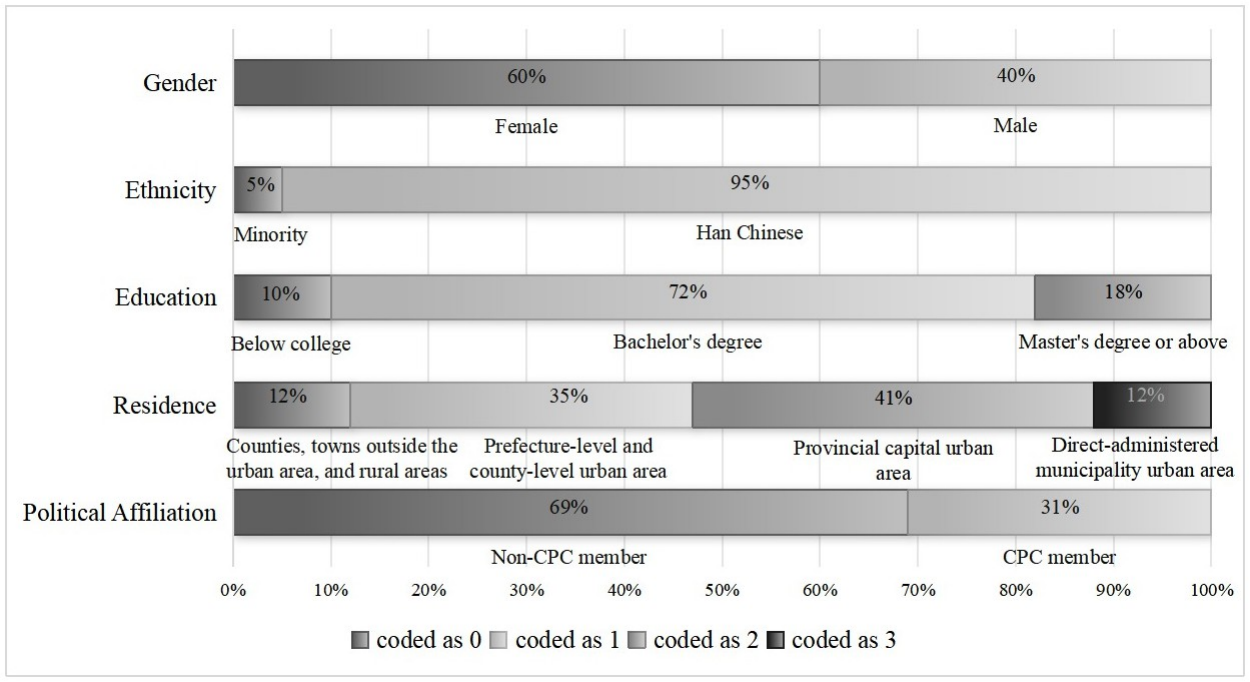
Characteristics of the participants.

Table 1 presented the descriptive statistical analysis of the research variables. Preliminary analysis regarding social confidence and social media exposure was shown in Fig 1. It revealed that respondents’ scores on social confidence exceeded the median of 3.5, and future confidence scores were significantly higher than present confidence scores (*t* = 2.451, *p*<0.05). This indicated that the public was confident in the current social development in China, particularly exhibiting positive expectations for the future. Regarding social media exposure, respondents generally reported frequent usage (M = 4.843, Std = 0.430), especially in the case of WeChat (*t* = 36.038, *p*<0.01). Among all surveyed samples (see Fig 2), 40% of respondents were male, with an average age of 32 years. Han Chinese constituted 95% of the respondents, and the majority had attained a bachelor’s degree (72%). Concerning residence, the highest proportion of respondents resided in provincial capital urban areas (41%), followed by prefecture-level and county-level urban areas (35%), while respondents from direct-administered municipalities and areas below the county level were less represented (both accounting for 12%). CPC members made up 31% of the respondents, and income and subjective social status were concentrated at a moderately high level (M = 3.892, M = 5.952).

**Table 1:** Descriptive statistical analysis of research variables.

	Variable	Mean	Std	Min	Max
Dependent variables	Social confidence (overall)	4.943	0.643	1.500	6
	Present confidence	4.927	0.713	1.250	6
	Future confidence	4.974	0.715	1.500	6
Independent variables	Social media exposure	4.843	0.430	2	5
	WeChat exposure	4.660	0.531	2	5
	Weibo exposure	3.626	0.926	1	5
Mediating variables	Group efficacy	4.255	0.411	1	5
	Group cohesion	4.323	0.395	1	5
Control variables	Gender	0.397	0.490	0	1
	Age	31.511	7.500	14	59
	Ethnicity	0.952	0.213	0	1
	Education	1.083	0.524	0	2
	Income	3.892	1.587	1	6
	Residence	1.534	0.864	0	3
	Political affiliation	0.308	0.462	0	1
	Subjective social status	5.952	1.392	1	10
Potential channel variables	Media trust	4.282	0.645	1	5
	Social capital	3.950	1.011	2	6
	Sense of social fairness	3.907	0.814	1	5

### Multivariate linear regression analysis

Table 2 reported the results of the baseline model using multivariate linear regression analysis. The data indicated that social media exposure had a significant positive impact on overall social confidence (*β* = 0.222, *p*<0.01), present confidence (*β* = 0.208, *p*<0.01), and future confidence (*β* = 0.251, *p*<0.01). Therefore, H1 was supported.

**Table 2:** Multivariate linear regression analysis.

Variable	Social confidence		
	Overall confidence	Present confidence	Future confidence
	Model 1–1	Model 1–2	Model 1–3
Gender	0.082** (0.036)	0.109*** (0.040)	0.028 (0.042)
Age	0.010*** (0.003)	0.008*** (0.003)	0.016*** (0.003)
Ethnicity	-0.172** (0.074)	-0.176** (0.079)	-0.166* (0.099)
Education (reference group: below college)			
Bachelor’s degree	0.008 (0.067)	0.003 (0.075)	0.017 (0.077)
Master’s degree or above	-0.130 (0.081)	-0.110 (0.089)	-0.170* (0.091)
Income	0.081*** (0.017)	0.114*** (0.019)	0.015 (0.019)
Residence (reference group: areas below the county level)			
Prefecture-level and county-level urban area	0.056 (0.066)	0.059 (0.075)	0.050 (0.072)
Provincial capital urban area	0.049 (0.068)	0.053 (0.076)	0.042 (0.076)
Direct-administered municipality urban area	-0.142* (0.080)	-0.127 (0.088)	-0.173* (0.093)
Political affiliation	-0.010 (0.035)	-0.006 (0.040)	-0.017 (0.041)
Subjective social status	0.078*** (0.017)	0.070*** (0.019)	0.094*** (0.019)
Social media exposure	0.222*** (0.045)	0.208*** (0.049)	0.251*** (0.055)
Constant	2.890*** (0.265)	2.933*** (0.294)	2.804*** (0.313)
Number of obersvations	1175	1175	1175
*R* ^*2*^	0.165	0.162	0.107

Note: Significance levels:

***, **, and * represent 1%, 5%, and 10%, respectively. Values in parentheses are standard errors. *VIF*_*max*_ = 3.12, *VIF*_*mean*_ = 1.85 indicate no multicollinearity issues.

Social confidence was also influenced by individual characteristics: (a) compared to females, males exhibited higher present confidence (*β* = 0.109, *p*<0.01) and overall confidence (*β* = 0.082, *p*<0.05), but no significant difference in future confidence (*β* = 0.028, n.s.). This could be attributed to the tendency of females to be more risk-averse, making them more sensitive to the threats and losses posed by current social issues, thereby weakening their present confidence. (b) Social confidence was higher among ethnic minorities than the Han Chinese (*β* = -0.172, *p*<0.05), benefiting from the ideological guidance of the Chinese National Community and the rapid development in minority regions. (c) As age (*β* = 0.010, *p*<0.01), income level (*β* = 0.081, *p*<0.01), and subjective social status (*β* = 0.078, *p*<0.01) increased—consistent with intuitive expectations—public social confidence became more positive. However, future confidence was influenced by subjective social status (*β* = 0.094, *p*<0.01) rather than objective income level (*β* = 0.015, n.s.), possibly due to perceived uncertainty about future income or the difficulty in assessing the adaptability of current income levels to future society. (d) Residents in direct-administered municipality urban areas exhibited lower future confidence (*β* = -0.173, *p*<0.1) and overall confidence (*β* = -0.142, *p*<0.1) compared to those in areas below the county level, while residents in other urban areas show no significant differences (*β* = 0.056, n.s.; *β* = 0.049, n.s.). A reasonable inference is that, despite the apparent geographical and socio-economic advantages of direct-administered municipalities, higher population/resource pressure, living costs, and social competition may increase the life pressure of residents, weakening their positive social confidence. As urbanization progresses, areas outside direct-administered municipalities maintain relatively stable living environments, social atmospheres, and resource allocations, resulting in smaller differences in both societal situation and expectations. Therefore, residents in these areas exhibit less disparity in social confidence compared to those in direct-administered municipalities.

Additionally, certain demographic characteristics had no significant impact on social confidence. (a) Concerning education, no significant differences in social confidence were found between "bachelor’s degree" and "master’s degree or above" compared to "below college" (*β* = 0.008, n.s.; *β* = -0.130, n.s.). Although higher education levels implies a higher likelihood of self-confidence, personal success, and positive future expectations, they may have also entailed greater societal expectations and pressures, potentially weakening the influence of education level on social confidence. Another possibility is that some individuals attributed their access to the highest level of education to their own efforts and ambitions while overlooking the significant role of social support systems such as social welfare and government policies. (b) Regarding political affiliation, it did not significantly influence social confidence (*β* = -0.010, n.s.). This suggests that the difference between CPC members and non-CPC members lies only in political identity rather than political identification. Especially with the development of online media and the establishment of official accounts by government and relevant administrative departments, the public has more direct and indirect channels for informal political participation. This has, to some extent, increased the understanding and identification of non-CPC members with the ruling party and the state, thereby weakening the confidence difference between them and party members.

### Robustness check

#### Dependent variable replacement

Social confidence encompasses a dual understanding from both a temporal and an event-based perspective. Considering that the temporal logic of social confidence underlines the characteristics of temporality and comprehensive evaluation, we prefer to embrace this perspective. Subsequently, in the robustness check, we employed event-based logic and the associated measurement method to substitute the dependent variable. Referring to the study by Zhang et al. [8], overall social confidence (M = 4.086, Std = 0.537, *α* = 0.918) comprised personal-event confidence (M = 4.089, Std = 0.576, *α* = 0.863) and societal-event confidence (M = 4.084, Std = 0.567, *α* = 0.859). The former included 10 items such as personal income, family economic status, housing conditions, health status, job situation, living conditions, family relationships, interpersonal relationships, social status, and development opportunities. The latter encompassed 11 items related to social atmosphere, employment opportunities, social fairness, food safety, public security conditions, social security level, medical service level, education level, price level, infrastructure, and environmental quality. Ratings were conducted using a five-point Likert scale. According to the results presented in Table 3, social media exposure continues to have a positive influence on social confidence (*β* = 0.082, *p*<0.05; *β* = 0.091, *p*<0.01; *β* = 0.074, *p*<0.1), indicating its constructive role in fostering social confidence. This finding further supports H1 and is consistent with the main research results.

**Table 3:** Regression analysis with dependent variable replacement.

Variable	Social confidence		
	Overall confidence	Personal-event confidence	Societal-event confidence
	Model 2–1	Model 2–2	Model 2–3
Social media exposure	0.082**	0.091***	0.074*
*R* ^*2*^	0.328	0.359	0.237

Note: *VIF*_*max*_ = 3.12, *VIF*_*mean*_ = 1.85 indicate no multicollinearity issues.

#### Independent variable replacement

In the previous analysis, this study used the frequency of social media exposure as a measure. However, there might be measurement errors associated with individuals’ subjectively perceived exposure frequency. Therefore, objective measurements were employed as a substitute for the independent variable. Respondents were asked, "On average, how much time do you spend on social media/ internet per day in the past week?" The ratio of the duration of the two was used as an improved measure of social media exposure (M = 0.650, Std = 0.333). The results in Table 4 indicate that the positive correlation between social media exposure and social confidence remains unchanged, whether considering social confidence from a temporal perspective (*β* = 0.107, *p*<0.05; *β* = 0.099, *p*<0.1; *β* = 0.125, *p*<0.05) or from an event perspective (*β* = 0.099, *p*<0.01; *β* = 0.078, *p*<0.05; *β* = 0.119, *p*<0.01). This suggests that the findings regarding H1 exhibit good robustness.

**Table 4:** Regression analysis with independent variable replacement.

Variable	Social confidence					
	Overall confidence (temporal)	Present confidence	Future confidence	Overall confidence (event-based)	Personal-event confidence	Societal-event confidence
	Model 3–1	Model 3–2	Model 3–3	Model 4–1	Model 4–2	Model 4–3
Social media exposure	0.107**	0.099*	0.125**	0.099***	0.078**	0.119***
*R* ^*2*^	0.147	0.150	0.089	0.328	0.357	0.239

Note: *VIF*_*max*_ = 3.12, *VIF*_*mean*_ = 1.84 indicate no multicollinearity issues.

#### Propensity score matching

To address endogeneity issues arising from self-selection bias, this study employed Propensity Score Matching (PSM) as a robustness check. The specific principles and steps included: (a) Division into low-level (control group) and high-level social media exposure (treated group) based on its mean. (b) Fitting a binary logit regression model to estimate the probability of high-level social media exposure based on observable characteristics, and obtaining propensity scores from this model. Subsequently, matching was performed on the research sample based on these obtained scores, ensuring comparable observable characteristics between the control and treated groups. (c) Repeating the main study based on the matching results. As shown in Table 5, the positive impact of social media exposure remained supported, supporting social confidence both from a temporal perspective (*β* = 0.217, *p*<0.01; *β* = 0.218, *p*<0.01) and from an event perspective (*β* = 0.078, *p*<0.05; *β* = 0.080, *p*<0.05), as well as their respective sub-dimensions. The research findings for H1 remain relatively stable.

**Table 5:** Propensity score matching analysis.

**Variable**	**Social confidence (temporal)**		**Present confidence**		**Future confidence**	
	**Radius-Matching**	**Kernel-Matching**	**Radius-Matching**	**Kernel-Matching**	**Radius-Matching**	**Kernel-Matching**
Social media exposure	0.217***	0.218***	0.207***	0.204***	0.237***	0.245***
*R* ^*2*^	0.153	0.153	0.153	0.152	0.095	0.097
**Variable**	**Social confidence (event-based)**		**Personal-event confidence**		**Societal-event confidence**	
	**Radius-Matching**	**Kernel-Matching**	**Radius-Matching**	**Kernel-Matching**	**Radius-Matching**	**Kernel-Matching**
Social media exposure	0.078**	0.080**	0.089***	0.089***	0.069*	0.072*
*R* ^*2*^	0.307	0.309	0.339	0.340	0.214	0.216

Note: The sample size for radius matching is 1124, and for kernel matching is 1136. For Radius-Matching models, *VIF*_*max*_ = 3.18, *VIF*_*mean*_ = 1.86; for Kernel-Matching models, *VIF*_*max*_ = 3.18, *VIF*_*mean*_ = 1.87. Those indicate no multicollinearity issues.

#### Nonlinear relationship testing

To explore the potential nonlinear relationship between social media exposure and social confidence, this study included the square term of social media exposure in the baseline model. The results from Table 6 revealed that the square term of social media exposure did not significantly impact social confidence from either a temporal perspective (*β* = 0.031, n.s.; *β* = -0.013, n.s.; *β* = 0.118, n.s.) or an event-based perspective (*β* = -0.043, n.s.; *β* = -0.060, n.s.; *β* = -0.026, n.s.). These findings indicated that a nonlinear relationship was not supported. The uniformity in public engagement with social media due to its widespread usage contributed to a consistent direction of influence on social confidence, thus strengthening the robustness of the linear relationship between the two variables.

**Table 6:** Non-linear relationship analysis.

**Variable**	**Social confidence (temporal)**	**Present confidence**	**Future confidence**
Social media exposure_Square	0.031	-0.013	0.118
Social media exposure	0.260***	0.192*	0.396***
*R* ^*2*^	0.165	0.162	0.109
**Variable**	**Social confidence (event-based)**	**Personal-event confidence**	**Societal-event confidence**
Social media exposure_Square	-0.043	-0.060	-0.026
Social media exposure	0.029	0.017	0.041
*R* ^*2*^	0.329	0.360	0.236

Note: *VIF*_*max*_ = 5.40, *VIF*_*mean*_ = 2.46 indicate no multicollinearity issues.

### Mediation analysis of collective efficacy

To strengthen the causal relationship between social media exposure and social confidence, we conducted an in-depth analysis of the logical formation of social confidence and made inferences about the mediating effect of collective efficacy. The specific methods and procedures are as follows: First, adopting Baron and Kenny’s stepwise approach [63], the first step examined the total effect (*α*_*1*_) of social media exposure on social confidence, and the second step tested the significance of the indirect effect (*γ*_*1*_*β*_*2*_), which means the amount of mediation. Second, considering the low testing power caused by the stepwise approach’s sequential tests of *γ*_*1*_ and *β*_*2*_, as well as the often unmet assumption of normal distribution, the bootstrap approach proposed by Preacher and Hayes [64] was employed. Finally, in reference to the reflections on the application of mediation analysis by Jiang [65], it was acknowledged that the effect estimates of the above two approaches based on linear regression tests might be biased. This bias could arise from potential confounding factors that simultaneously influence collective efficacy and social confidence. For example, in the case of public trust in the government, individuals who trust the government are likely to obtain public affairs information from both the government and its affiliated media outlets, thereby enhancing their sense of collective efficacy. Simultaneously, they are more inclined to positively acknowledge the government’s performance, contributing to a boost in overall social confidence [12, 66]. Additionally, the bias also arise from a potential reciprocal causal relationship between collective efficacy and social confidence, where social confidence, as a social psychological resource, could in turn enhance collective efficacy. Given the potential estimation bias, we further employed a Channel Testing of Causality to provide exploratory evidence for the mediating role of collective efficacy.


$$Y=\alpha_{0}+\alpha_{1}D+\varepsilon_{Y_{1}}$$ (2)

$$M=\gamma_{0}+\gamma_{1}D+\varepsilon_{M}$$ (3)

$$Y=\beta_{0}+\beta_{1}D+\beta_{2}M+\varepsilon_{Y_{2}}$$ (4)

Table 7 presented the results of the stepwise mediation analysis. Model 5–2 and 5–3 revealed that social media exposure significantly and positively influenced group efficacy (*β* = 0.084, *p*<0.05) and group cohesion (*β* = 0.222, *p*<0.01). Model 5–4 and 5–5 showed that, after controlling for social media exposure, group efficacy (*β* = 0.676, *p*<0.01) and group cohesion (*β* = 0.698, *p*<0.01) both had a significant and positive impact on social confidence. Additionally, the coefficients associated with social media exposure were smaller than the total effect coefficient observed in Model 5–1. This suggested that both group efficacy and group cohesion can play a mediating role between social media exposure and social confidence. These findings provided preliminary support for H3a and H3b.

**Table 7:** Stepwise mediation analysis.

Variable	Social confidence	Group efficacy	Group cohesion	Social confidence	Social confidence
	Model 5–1	Model 5–2	Model 5–3	Model 5–4	Model 5–5
Social media exposure	0.222***	0.084**	0.222***	0.166***	0.067
Group efficacy				0.676***	
Group cohesion					0.698***
*R* ^*2*^	0.165	0.119	0.161	0.329	0.319

Note: For Model 5–1 to Model 5–5, *VIF*_*max*_ = 3.13 indicate no multicollinearity issues.

The bootstrap mediation analysis provided direct access to the bootstrap distribution of the indirect effect, allowing for the calculation of standard errors and confidence intervals for the indirect effect. As shown in Table 8, the indirect effects of group efficacy (*β* = 0.057, *p*<0.05, 95% CI: 0.008–0.105, not including 0) and group cohesion (*β* = 0.155, *p*<0.01, 95% CI: 0.090–0.220, not including 0) as mediating variables were both significant and smaller than the total effect values. This further validated the explanatory power of group efficacy and group cohesion in the process through which social media exposure influenced social confidence. By comparing the magnitudes of the indirect effects, it was evident that group cohesion played a more significant mediating role in this process compared to group efficacy.

**Table 8:** Bootstrap mediation analysis.

		Social media exposure→social confidence				
**Mediating effect**		**Model 6–1**				
		**Β**	**Se**	**P>|z|**	**CI**	
Group efficacy	Indirect effect	0.057**	0.025	0.022	0.008	0.105
	Direct effect	0.166***	0.046	0.000	0.076	0.255
	Total effect	0.222***	0.045	0.000	0.133	0.311
**Mediating effect**		**Model 6–2**				
		**Β**	**Se**	**P>|z|**	**CI**	
Group cohesion	Indirect effect	0.155***	0.033	0.000	0.090	0.220
	Direct effect	0.067	0.045	0.138	-0.022	0.156
	Total effect	0.222***	0.047	0.000	0.129	0.315

The Channel Testing of Causality helps to better understand the causal relationship between social media exposure and social confidence in terms of explanatory significance and provides support for the identification of the mediating effect of collective efficacy. The principles and steps are as follows: First, examine the effect of social media exposure on social confidence (*α*_*1*_ in Eq (2)), second, re-examine this effect after incorporating the channel variable (*β*_*1*_ in Eq (4)), and finally, compare the numerical changes between the two, revealing the role of channel variables in explaining the impact of social media exposure on social confidence. Table 9 reported the results of the channel approach. Comparing Model 7–1 and 7–2, it was found that the estimated coefficient of social media exposure on social confidence decreased significantly from 0.222 to 0.166, with a reduction of 25.23% (i.e., 1—*β*_*1*_/*α*_*1*_). Similarly, according to Model 7–3 to 7–6, after incorporating group cohesion, media trust, social capital, and the sense of social fairness into the model, the reduction percentages of the social media exposure coefficient were 69.82%, 40.54%, 3.60%, and 16.67%, respectively. This indicated that group cohesion, media trust, and collective efficacy can cause larger changes in the coefficient of the impact of social media exposure on social confidence, playing relatively important mediating roles in this process. Particularly, the combination of Model 7–3 and 7–7 showed that the social media exposure coefficient became nonsignificant after controlling for group cohesion, and there was no significant difference compared to the coefficient after controlling for all channel variables, highlighting the crucial role of group cohesion as the most important channel through which social media builds social confidence.

**Table 9:** Results of channel testing of causality.

Variable	Social confidence						
	Model 7–1	Model 7–2	Model 7–3	Model 7–4	Model 7–5	Model 7–6	Model 7–7
Social media exposure	0.222***	0.166***	0.067	0.132***	0.214***	0.185***	0.059
Group efficacy		0.676***					0.276***
Group cohesion			0.698***				0.300***
Media trust				0.450***			0.264***
Social capital					0.070***		0.010
Sense of social fairness						0.352***	0.192***
*R* ^*2*^	0.165	0.329	0.319	0.350	0.174	0.323	0.495

Note: For Model 7–1 to Model 7–7, *VIF*_*max*_ = 3.14 indicate no multicollinearity issues.

### Social media exposure heterogeneity analysis

In the preceding analysis, the causal relationship and mediating channels between social media exposure and social confidence have been clarified. It is essential to further examine the social confidence effects of heterogeneous social media exposure. According to the results presented in Table 10, both WeChat exposure (*β* = 0.067, *p*<0.05) and Weibo exposure (*β* = 0.085, *p*<0.01) positively influenced social confidence. The positive effect of WeChat exposure was more pronounced in future confidence (*β* = 0.104, *p*<0.05), while the positive effect of Weibo exposure was significant in both present confidence and future confidence (*β* = 0.071, *p*<0.01; *β* = 0.113, *p*<0.01). At this point, H2a was partially supported, and H2b was fully supported.

**Table 10:** Results of the impact of heterogeneous media exposure.

Variable	Social confidence		
	Overall confidence	Present confidence	Future confidence
	Model 8–1	Model 8–2	Model 8–3
WeChat exposure	0.067**	0.048	0.104**
Weibo exposure	0.085***	0.071***	0.113***
*R* ^*2*^	0.163	0.158	0.116

Note: *VIF*_*max*_ = 3.15, *VIF*_*mean*_ = 1.80 indicate no multicollinearity issues.

This study then employed propensity score matching to examine the heterogeneous media exposure effects, ensuring the robustness of the findings mentioned above. As presented in Table 11, WeChat exposure significantly influenced overall confidence (*β* = 0.065, *p*<0.05; *β* = 0.067, *p*<0.05) and future confidence (*β* = 0.104, *p*<0.05; *β* = 0.104, *p*<0.05), but did not significantly affect present confidence (*β* = 0.046, n.s.; *β* = 0.048, n.s.). Meanwhile, Weibo exposure had a significant impact on overall confidence (*β* = 0.087, *p*<0.01; *β* = 0.082, *p*<0.01), present confidence (*β* = 0.073, *p*<0.01; *β* = 0.073, *p*<0.01), and future confidence (*β* = 0.115, *p*<0.01; *β* = 0.116, *p*<0.01), consistent with the aforementioned research findings.

**Table 11:** Propensity score matching analysis.

Variable	Overall confidence		Present confidence		Future confidence	
	Radius-Matching	Kernel-Matching	Radius-Matching	Kernel-Matching	Radius-Matching	Kernel-Matching
WeChat exposure	0.065**	0.067**	0.046	0.048	0.104**	0.104**
Weibo exposure	0.087***	0.082***	0.073***	0.073***	0.115***	0.116***
*R* ^*2*^	0.161	0.163	0.155	0.157	0.114	0.115

Note: The sample size for radius matching is 1165, and for kernel matching is 1172. For Radius-Matching models, *VIF*_*max*_ = 3.19, *VIF*_*mean*_ = 1.82; for Kernel-Matching models, *VIF*_*max*_ = 3.16, *VIF*_*mean*_ = 1.81. Those indicate no multicollinearity issues.

The above results revealed differences in the components of social confidence arising from heterogeneous media exposure, confirming the multilateral relationship between social media exposure and social confidence, a phenomenon known as the differentiated impact of heterogeneous social media exposure on social confidence [5]. To gain a deeper understanding of this impact, we further explored the distinct pathways through which heterogeneous social media exposure influences social confidence. The results presented in Table 12 indicated that, regarding the impact process of WeChat exposure on social confidence, only the indirect path through group cohesion was significant (95% CI: 0.025–0.089, not including 0). In contrast, for Weibo exposure, both indirect paths through group efficacy (95% CI: 0.013–0.058) and group cohesion (95% CI: 0.018–0.061) were significant, with neither interval including 0. This verification suggested that there were differentiated pathways in the impact of heterogeneous social media exposure on social confidence.

**Table 12:** Mediation analysis of heterogeneous media exposure (Bootstrap method).

		WeChat exposure→social confidence				Weibo exposure→social confidence			
**Mediating effect**		**Model 9–1**				**Model 9–2**			
		**Β**	**P>|z|**	**CI**		**Β**	**P>|z|**	**CI**	
Group efficacy	Indirect effect	0.027*	0.090	-0.004	0.059	0.036***	0.002	0.013	0.058
	Direct effect	0.040	0.236	-0.026	0.105	0.050**	0.012	0.011	0.088
	Total effect	0.067**	0.048	0.000	0.133	0.085***	0.000	0.043	0.127
**Mediating effect**		**Model 9–3**				**Model 9–4**			
		**Β**	**P>|z|**	**CI**		**Β**	**P>|z|**	**CI**	
Group cohesion	Indirect effect	0.057***	0.000	0.025	0.089	0.040***	0.000	0.018	0.061
	Direct effect	0.010	0.761	-0.054	0.073	0.046**	0.026	0.005	0.086
	Total effect	0.067**	0.040	0.003	0.131	0.085***	0.000	0.042	0.129

## Conclusion and discussion

### Conclusions

This study, grounded in the framework of Media-system Dependency Theory, systematically examined the impact of social media exposure on social confidence and its underlying mechanisms. The key findings are as follows:

Firstly, social media exposure positively promotes social confidence. Even after robustness checks such as variable replacement and propensity score matching, this positive effect remains confirmed. The results are consistent with those of Zhang et al. [11] and Pan and Luo [17], underscoring the reliability of the positive relationship between social media and social confidence within the Chinese context. However, whether this relationship is applicable in Western countries is questionable. While direct research linking social media to social confidence in Western contexts is scarce, some scholars have noted that internet exposure negatively impacts interpersonal trust and social confidence [5]. In China, official media representing the party and government timely regulate and guide public opinion trends, suppressing discussions on sensitive political topics while avoiding the emergence of extreme emotions. In contrast, in certain Western countries, values of individualism and liberalism foster more open dialogues, rendering political differences or social conflicts more pronounced or even amplified through social media. This may be the underlying reason for the speculation regarding the negative effects of social media.

Secondly, the influence of social media exposure on social confidence operates through the mechanism of collective efficacy. Group efficacy and group cohesion, focusing on the understanding domains of "efficacy" and "collective," respectively, are regarded as independent and distinct emergent states within the group. Considering them as independent explanatory variables, their explanatory power in the process of social media exposure influencing social confidence has been verified. Notably, the results of the Channel Testing of Causality highlighted that group cohesion is the most crucial channel through which social media exposure affects social confidence. This supports the perspective of David Holmes, who, from a "ritual" standpoint, contends that the primary function of media is not merely to convey information and serve individual interests but to aggregate a broad audience into some form of community, providing them with a sense of belonging.

Thirdly, the differentiated impact of heterogeneous social media exposure on social confidence does not manifest in a consistent direction of influence but rather in the components of social confidence and the influence pathways. Both Wechat and Weibo exposure propel positive developments in social confidence. For the former, this driving force is more evident in the future aspect of social confidence and requires the influence pathway of group cohesion. As for the latter, such media exposure is meaningful for both present and future confidence, with group efficacy and group cohesion serving as effective pathways in this influencing process.

Specifically, WeChat and Weibo both navigate the intricate relationship between social media and social confidence. They can exert both positive and negative influences, yet generally tend to have a positive impact influenced by China’s collectivist culture and political system. On one hand, in WeChat, based on strong ties, the public tends to self-regulate. In contrast, on Weibo, characterized by weak ties, discussions on more diverse topics and less conservative public opinions may undermine social confidence. However, the social understanding and civic responsibility fostered by a relatively free speech environment still contribute positively to social confidence on both platforms. On the other hand, while some self-media outlets may manipulate public opinion and spread rumors for commercial gain, official media, leveraging their credibility, can govern public opinion and promote positive public confidence. This is because in China, media credibility assessment includes not only the professional dimension but also the power dimension, with the latter emphasizing official status, administrative level, and the official nature of content as decisive factors in determining authority [37, 43]. Furthermore, both WeChat and Weibo operate within the framework of their political attributes and support for the power system, ensuring that all content aligns with political guidelines [43]. Therefore, after examining the user composition of WeChat and Weibo, it is concluded that both platforms still play a positive role in influencing social confidence.

According to the Strength of Weak Ties [67], Weibo’s weak ties can bridge different social groups, facilitating the flow of information, ideas, and beliefs across a wider range. This flow not only provides broader social resources and opportunities for public social mobility, enhancing the perception of possibilities for change in the current situation and the future but also breaks down the limitations and barriers of different social circles, promoting social inclusivity and diversity. Therefore, group efficacy and cohesion are feasible pathways for weak-tie social media to construct social confidence. In contrast, WeChat’s strong ties to some extent confine social circles to offline social networks, leading to concerns about personal identity recognition among the public. This limitation may restrict free speech on social issues and even the willingness to propose suggestions [33]. Although WeChat may exhibit positive representations of societal attitudes overall, it may actually obscure social conflicts and negative public opinion, hindering the actual resolution of current societal issues. This is because strong-tie social media has no significant impact on group efficacy and present confidence.

### Theoretical implications

Firstly, this paper contributes to a deeper understanding of the conceptual essence and scientific measurement of social confidence, while also highlighting the role of social media in promoting it. The study starts by addressing past research’s incomplete understanding of social confidence. Some studies have overemphasized the present dimension of social confidence, treating social trust or present confidence as synonymous with overall social confidence, while neglecting its temporality. Additionally, the use of single or limited indicators to represent social confidence overlooks its multifaceted nature. For instance, equating public attitudes towards government or the economy with overall social confidence fails to consider its diverse event components. Building upon these considerations, this paper comprehensively understands the scientific connotation of social confidence and utilizes mature systematic measurement while fully considering its temporal characteristics and diverse event composition. It affirms the positive constructive role of social media on social confidence from both temporal (including present and future confidence) and event perspectives (including personal-event and societal-event confidence). This not only deepens the understanding of social confidence and provides substantial evidence for the relationship between social media and it but also enriches relevant research on enhancing social confidence in virtual environments rather than real ones.

Secondly, this paper delves into the underlying logic behind the relationship between social media and social confidence, emphasizing the prominent mechanism of group cohesion and providing practical guidance for identifying key pathways in social confidence construction. While existing research extensively acknowledges the mediating role of perceived media trust in this relationship, few studies have examined alternative pathways or conducted comparative analyses. Recognizing social media’s role in facilitating networked relationships among society members, this paper adopts the perspective of "individuals within the group." It first validates the mechanisms of group efficacy and cohesion in constructing social confidence through social media. Subsequently, utilizing the Channel Testing of Causality method, it compares and identifies potential pathways, highlighting the importance of group cohesion, perceived media trust, and group efficacy channels, with particular emphasis on the explanatory power of group cohesion. This research perspective addresses limitations in previous studies, which solely focused on individual cognition or experience as the driving factors of social psychology, underscoring the significance of connections, solidarity, and consensus among individuals in social confidence construction. It offers valuable theoretical and methodological guidance for future exploration in this area.

Thirdly, this paper enhances our grasp of the complex multilateral relationship between heterogeneous social media exposure and social confidence, adding depth to the research framework concerning how social media constructs social confidence. While prior studies comparing the impacts of traditional and electronic media on social confidence have identified the multilateral relationship between media exposure and social confidence, they have predominantly focused on the extent of media influence. Moreover, they have overlooked the multilateral relationship between different types of media within the same category and social confidence. However, this study uncovers that the varying impacts of platforms like WeChat and Weibo on social confidence are not manifested in main effects but rather in diverse components of social confidence and various pathways of influence. These findings significantly contribute to our comprehension of how social media exposure influences social confidence, paving the way for a more holistic understanding of the intricate multilateral relationship between media and social confidence.

### Practical implications

Firstly, there is a need for a profound understanding of the multifaceted functional roles that social media plays in constructing social confidence. Individuals are becoming increasingly proactive in expressing public opinions and showcasing societal attitudes on social media platforms, making social confidence more visibly tangible. Simultaneously, this study reveals the significant importance in establishing, aggregating, and boosting social confidence. The government should reinforce the trend towards intelligent governance and enhance the level of intelligent governance. They should leverage emerging information technologies to promptly, accurately, and dynamically grasp online public sentiment and public mentality, establishing a robust monitoring and early warning system for societal psychology. Moreover, governments and relevant regulatory authorities need to effectively utilize social media platforms to disseminate information on public concerns such as healthcare and legal regulations, promptly share policy initiatives, work plans, and achievements with the public to maintain government openness and transparency, and shape a positive government image. These measures will provide active psychological resources and mental support for the sustained and stable operation of the social system.

Furthermore, it is crucial to recognize the significant importance of managing societal emotions and improving psychological expectations to boost public confidence. This stems from the findings of this study, which reveal that social media exposure affects both present and future confidence. Given the inevitable contradictions and conflicts in the process of societal development, it is crucial to prevent the accumulation of hostile emotions during conflicts. Social media, acting as a safety valve for society, should timely release, guide, and intervene in public emotions. This can be achieved by actively guiding reasonable speech and rational empathy through online education or discussion forums, and by regularly collecting, listening to, and responding to public opinions and suggestions through official media accounts. Moreover, efforts to clarify rumors, dispel misunderstandings, establish interactive relationships, and mutual trust between the government and the public are essential for seeking a harmonious coexistence between official public opinion and grassroots public opinion within the same media space. Additionally, social media platforms need to fully acknowledge their social responsibility by enhancing the discernment, regulation, and auditing capabilities of information content, ensuring that the public not only has an accurate understanding of the current development situation in the country but also comprehends the future policy intentions of the party and the government.

Lastly, considering the significant role of social cohesion channels, it is essential to value and leverage the responsibility and influence of social media in fostering consensus and cohesion. Social media subtly influences people’s daily lives and thought patterns; therefore, achieving consensus on societal norms, values, and culture requires joint efforts from the government and the media. The government can utilize social media platforms to strengthen networking between individuals and different groups, facilitating mutual understanding, shared cognition, and consensus on common positions and beliefs. For instance, creating groups for hot public events can stimulate free dialogue and experience sharing among the public. Special attention should be given to preventing the Balkanization of the Internet, thus avoiding consensus dilemmas that may arise from information gaps between groups. Social media platforms should regulate and balance various public issues in the online environment, bridging differences in opinions and perceptions from the stage of information exposure, and achieving a collective commitment for unity and progress among all members of society, thus consolidating the strength for Chinese-style development.

### Research limitations and future prospects

This paper has certain limitations and shortcomings, and based on this, future research prospects are proposed. Firstly, when considering the continuity of time and the relative stability of social confidence, people’s present confidence is also based on past social performance and expectations. The cross-sectional data in this paper makes it impossible to discuss the antecedents and processes of social confidence formation in a complete time framework of "past-present-future." Future research could address this limitation by selecting a relatively stable group of respondents and implementing periodic survey and data collection plans to track their levels of social confidence at different time points. This would allow for the observation of the developmental trajectory and dynamic changes in social confidence over time. Moreover, by lagging one period of social confidence, these data could be incorporated into empirical models using dynamic panel data to test the reliability of research results.

Secondly, people’s social cognition is derived from both their personal observations and experiences of society and the mimicry environment constructed by mass media. While this article focuses on social media’s role in shaping social confidence, it does not delve into the impact of individual experiences of societal reality or their combined effects. For example, when individuals encounter unexpected public events, whether the expectations of a better world created by social media will bring comfort and hope or be hindered by personal hardships remains unexplored. Future research could further investigate the joint shaping role of social media and societal practice factors on social confidence. Specifically, incorporating individual experiences and government performance into research models and exploring their respective interactions with social media exposure could be fruitful. We speculate that social media exposure, representing the collective societal mindset, may lead individuals to attribute their predicaments or successes primarily to themselves rather than external societal factors, thus weakening the impact of individual experiences on social confidence. Conversely, social media may magnify the effects of government performance on social confidence, potentially leading to more extreme outcomes.

Thirdly, while this study has incorporated demographic characteristics into the research model and identified the influence of individual features, providing potential explanations for the results, it remains confined to descriptive phenomena and speculative causation. To further deepen theoretical reflections and provide empirical evidence, subsequent research could conduct face-to-face structured interviews with individuals of diverse demographic characteristics. These interviews would aim to uncover deeper insights into their intrinsic social beliefs, values, overall attitudes towards social structure, institutions, policies, social networks and relationships, and expectations for social change and development. Additionally, comparative analyses of social confidence representation across different demographic groups in future studies can offer a more comprehensive understanding of the differences and commonalities among different groups in the formation of social confidence.

### Concluding statement

In conclusion, this study significantly enhances our understanding of the role of social media in constructing social confidence within the Chinese context. Despite the concurrent influence of both positive and negative factors, the positive relationship between social media exposure and social confidence stands out, largely influenced by China’s collectivist culture and media political attributes. In this positive relationship, social media plays a pivotal role in fostering public cohesion and consensus-building, departing from previous focal points on factors like media trust and perceived efficacy. When considering heterogeneous social media, there exist more nuanced multilateral relationships between social media and social confidence. Notably, China’s two most prevalent social media platforms, Tencent WeChat and Sina Weibo, impact different components of social confidence and follow distinct pathways of influence. While WeChat, based on strong ties, tends to exhibit positive representations of social confidence, it falls short in imbuing the public with a sense of efficacy in addressing societal issues. Conversely, Weibo, based on weak ties, may feature more extreme opinion trends, yet the opportunities for free speech and spurred civic participation contribute to elevating social confidence.

## Supporting information

S1 Data (XLSX)

S1 File Questionnaire. (DOCX)
