# Supplementary material for: How social media exposure constructs social confidence: An empirical study on impact, mechanisms, and multilateral relationships
Source: PLoS One. 2024 Sep 17;19(9):e0308745. doi: 10.1371/journal.pone.0308745 (PMC11407681; doi:10.1371/journal.pone.0308745)
Supplement: S1 File — (DOCX) [file pone.0308745.s002.docx]

**社会心态情况调查**

**Survey on social attitudes**

您好！首先感谢您在百忙之中参与本次问卷调查。本次调查目的是了解大众社会心态基本情况，本调研采取无记名问卷形式，答案没有对错之分，请您结合实际情况认真填写，调研结果仅供研究使用，坚决不会泄露您的个人信息。未成年人请在监护人的陪同、监督和同意下完成该问卷。您的真诚合作对本研究非常重要，衷心感谢您的支持！

Hello! First of all, thank you for taking the time to participate in this survey. The purpose of this survey is to understand the basic situation of public social attitudes. This research is conducted in the form of an anonymous questionnaire, and there are no right or wrong answers. Please fill it out carefully based on your actual situation. The research results are for study purposes only and will not disclose your personal information. Minors, please complete this questionnaire under the accompaniment, supervision, and consent of your guardian. Your sincere cooperation is crucial to this study, and we sincerely appreciate your support!

| [按钮]： 确认 或者 退出  [Button]: Confirm or Exit |
| --- |

**‾ ‾ ‾ ‾ ‾ ‾ ‾ ‾ ‾ ‾ ‾ ‾ ‾ ‾ ‾ ‾ ‾ ‾ ‾ ‾ ‾ ‾ ‾ ‾ ‾ ‾ ‾ ‾ ‾ ‾ ‾ ‾ ‾ ‾ ‾ ‾ ‾ ‾ ‾ ‾ ‾ ‾ ‾ ‾ ‾ ‾ ‾ ‾ ‾ ‾ ‾ ‾ ‾ ‾ ‾ ‾ ‾ ‾ ‾ ‾ ‾ ‾ ‾**

**默认块-版块开始**

**Default Block - Block Start**

Q1 性别 [单选]


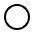
 男


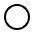
 女

Q1 Gender [Single choice]


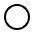
 Male


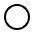
 Female

Q2 年龄 [填空]

Q2 Age [Fill in the blank]

Q3 民族 [单选]


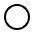
 汉族


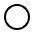
 少数民族

Q3 Ethnicity [Single choice]


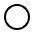
 Han Chinese


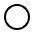
 Minority

Q4 最高教育程度 [单选]


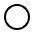
 小学及以下


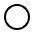
 初中


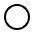
 高中/中专/技校


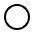
 大专


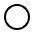
 本科


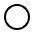
 硕士及以上

Q4 Highest education level [Single choice]


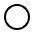
 Elementary school and below


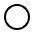
 Junior high school


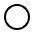
 Senior high school/ vocational school/ technical School


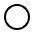
 College


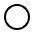
 Bachelor's degree


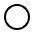
 Master's degree and above

Q5 月均收入 [单选]


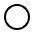
 0-3000元


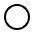
 3001-5000元


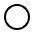
 5001-8000元


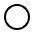
 8001-10000元


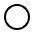
 10001-15000元


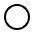
 15001-30000元


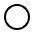
 30000元以上

Q5 Average monthly income [Single choice]


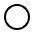
 0-3000 RMB


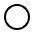
 3001-5000 RMB


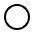
 5001-8000 RMB


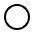
 8001-10000 RMB


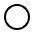
 10001-15000 RMB


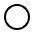
 15001-30000 RMB


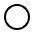
 Above 30000 RMB

Q6 居住地 [单选]


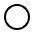
 直辖市城区


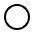
 省会城市城区


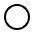
 地/县级市城区


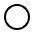
 县城


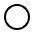
 市/县城城区以外的镇


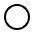
 农村

Q6 Residence [Single choice]


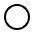
 Direct-administered municipality urban area


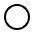
 Provincial capital urban area


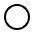
 Prefecture-level and county-level urban area


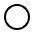
 County


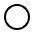
 Towns outside the urban area of the county or city


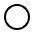
 Rural areas

Q7 居住地 [单选]


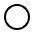
 东部


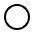
 中部


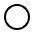
 西部


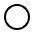
 东北

Q7 Residence [Single choice]


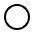
 Eastern


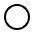
 Central


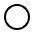
 Western


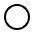
 Northeastern

Q8 政治面貌 [单选]


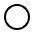
 中共党员


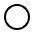
 非中共党员

Q8 Political affiliation [Single choice]


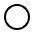
 Communist Party of China (CPC) member


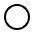
 Non-CPC member

Q9 在我们的社会里，有些人处在社会的上层，有些人处在社会的下层。综合看来，在目前这个社会上，您本人处于社会哪一层? [量表]

[最底层,最顶层]

Q9 In our society, some people are in the upper strata, while others are in the lower strata. Overall, in the current society, which social stratum do you personally believe you belong to? [Scale]

[Lower Stratum, Upper Stratum]


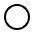
 1
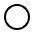
 2
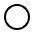
 3
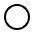
 4
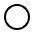
 5
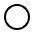
 6
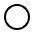
 7
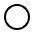
 8
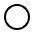
 9
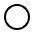
 10

Q10 您最近一周平均每天上网时间（小时）____ [填空]

Q10 Average daily internet usage in the past week (hours) ____ [Fill in the blank]

Q11 最近一周，平均每天使用社交媒体时间（小时）____。注：社交媒体包括微信、QQ、微博、博客、社区论坛、抖音、快手等。 [填空]

Q11 Average daily social media usage in the past week (hours) ____. Note: Social media includes WeChat, QQ, Weibo, blogs, community forums, TikTok, Kuaishou, etc. [Fill in the blank]

Q12 最近一周，平均每天使用传统媒体时间（小时）____。注：传统媒体包括报纸、杂志、广播、电视等。 [填空]

Q12 Average daily traditional media usage in the past week (hours) ____. Note: Traditional media includes newspapers, magazines, radio, television, etc. [Fill in the blank]

Q13 社交媒体使用频率 [单选]


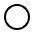
 从来不用


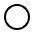
 每年数次


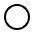
 每月数次


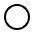
 每周数次


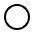
 每天数次

Q13 Social media usage frequency [Single choice]


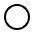
 Never use


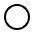
 Several times a year


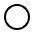
 Several times a month


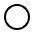
 Several times a week


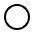
 Several times a day

Q14 微信使用情况 [单选]


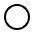
 从不


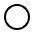
 很少


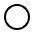
 有时


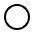
 经常


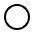
 非常频繁

Q14 WeChat usage frequency [Single choice]


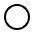
 Never


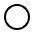
 Rarely


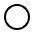
 Occasionally


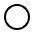
 Often


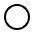
 Very frequently

Q15 微博使用情况 [单选]


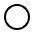
 从不


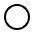
 很少


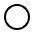
 有时


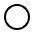
 经常


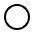
 非常频繁

Q15 Weibo usage frequency [Single choice]


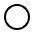
 Never


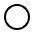
 Rarely


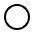
 Occasionally


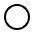
 Often


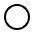
 Very frequently

Q16 一般情况下，从周一到周五，您每天通过网络联系的人大概有多少？ [单选]


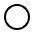
 没有


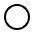
 0-4

5-9

10-19

20-49

50人及以上

Q16 On a typical weekday, how many people do you communicate with through the internet? [Single choice]

None

0-4

5-9

10-19

20-49

50 or more people

Q17 以下是有关官方新闻媒体的表述。请根据您的认同程度选择符合的选项。 [矩阵量表]

Q17 The following statements are related to official news media. Please choose the options that best represent your level of agreement. [Matrix scale]

|  | 完全不同意  Strongly disagree | 比较不同意  Somewhat disagree | 中立  Neutral | 比较同意  Somewhat agree | 完全同意  Strongly agree |
| --- | --- | --- | --- | --- | --- |
| 总的来说，我对官方新闻媒体是信任的  In general, I trust official news media |  |  |  |  |  |
| 内容真实，不含虚假、猜测和虚构成分  Content is truthful, without falsehood, speculation, or fabrication |  |  |  |  |  |
| 准确无误  Accurate and error-free |  |  |  |  |  |
| 报道完整，不回避重要事实  Reports are comprehensive, not avoiding important facts |  |  |  |  |  |
| 报道公正，不偏袒任何一方  Reports are impartial, not biased towards any party |  |  |  |  |  |
| 客观呈现事件原貌，不加入报道者偏见  Objectively presents the events as they are, without incorporating the reporter's bias |  |  |  |  |  |
| 尽可能报道一切民众想知道的事件  Reports everything that the public would want to know as much as possible |  |  |  |  |  |

Q18 您对以下表述的同意程度。 [矩阵量表]

Q18 Your level of agreement with the following statements. [Matrix scale]

|  | 完全不同意  Completely disagree | 比较不同意  Somewhat disagree | 中立  Neutral | 比较同意  Somewhat agree | 完全同意  Completely agree |
| --- | --- | --- | --- | --- | --- |
| 人们的集体行动对公共事务有很大影响  Collective actions have a significant impact on public affairs |  |  |  |  |  |
| 人们的集体行动能改善社会  Collective actions by people can improve society |  |  |  |  |  |
| 如果足够多的人要求改变，政府及相关部门会回应该需求  If enough people demand change, the government and relevant departments will respond to those demands |  |  |  |  |  |
| 如果足够多的人要求改变，政府及相关部门会采取措施  If enough people demand change, the government and relevant departments will respond to those demands |  |  |  |  |  |
| 我愿意帮助他人  I am willing to help others |  |  |  |  |  |
| 我与周围的人关系密切  I have close relationships with people around me |  |  |  |  |  |
| 我与周围的人相处和谐  I get along harmoniously with people around me |  |  |  |  |  |
| 我认为他人是可以信任的  I believe others can be trusted |  |  |  |  |  |
| 我愿意未来继续与周围的人保持联系  I am willing to stay in touch with people around me in the future |  |  |  |  |  |
| 群体应有清楚的行为规范  There should be clear behavioral norms in the group |  |  |  |  |  |
| 群体中成员应清楚自己职责、行为有序  Members in the group should be clear about their responsibilities, and behave orderly |  |  |  |  |  |

Q19 气候变化是当今重要的社会议题，也与每个公民息息相关。请问您对下述问题的同意程度如何。 [矩阵量表]

Q19 Climate change is an important social issue today and is closely related to every citizen. How much do you agree with the following statements? [Matrix scale]

|  | 完全不同意  Completely disagree | 比较不同意  Somewhat disagree | 无所谓同意不同意  Neutral/ no preference | 比较同意  Somewhat agree | 完全同意  Completely agree |
| --- | --- | --- | --- | --- | --- |
| 为气候变化做点事情是一个机会，让我们为国家事业做贡献  For taking action on climate change is an opportunity to contribute to the national cause |  |  |  |  |  |
| 人们可以对气候变化产生影响  People can have an impact on climate change |  |  |  |  |  |
| 气候变化的挑战将给人们带来使命感  The challenges of climate change will bring a sense of mission to people |  |  |  |  |  |
| 气候变化将促进更强的社区精神和联系  Climate change will foster a stronger sense of community spirit and connection |  |  |  |  |  |
| 我们在气候变化问题上无能为力  We are powerless in the face of climate change |  |  |  |  |  |

Q20 您是否从事过以下活动？ [矩阵量表]

Q20 Have you ever engaged in the following activities? [Matrix scale]

|  | 是  Yes | 否  No |
| --- | --- | --- |
| 您是否在现实社会中讨论过气候变化?  Have you discussed climate change in the real world? |  |  |
| 您是否在互联网上讨论过气候变化?  Have you discussed climate change on the internet? |  |  |
| 您是否在互联网上转发过与气候变化有关的信息?  Have you shared information related to climate change on the internet? |  |  |

Q21 本题检测是否认真作答，请选不满意。 [单选]

非常不满意

不满意

满意

非常满意

Q21 This question is to check if you are answering seriously, please choose ''dissatisfied.'' [Single choice]

Very dissatisfied

Dissatisfied

Satisfied

Very satisfied

| 条件： 非常不满意 未选定 跳至： 自动拒绝  Conditions: very dissatisfied, not selected, skip to: auto rejection |
| --- |

**‾ ‾ ‾ ‾ ‾ ‾ ‾ ‾ ‾ ‾ ‾ ‾ ‾ ‾ ‾ ‾ ‾ ‾ ‾ ‾ ‾ ‾ ‾ ‾ ‾ ‾ ‾ ‾ ‾ ‾ ‾ ‾ ‾ ‾ ‾ ‾ ‾ ‾ ‾ ‾ ‾ ‾ ‾ ‾ ‾ ‾ ‾ ‾ ‾ ‾ ‾ ‾ ‾ ‾ ‾ ‾ ‾ ‾ ‾ ‾ ‾ ‾ ‾**

Q22 总的来说，您认为当今的社会公不公平? [单选]

完全不公平

比较不公平

说不上公平但也不能说不公平

比较公平

完全公平

不知道

Q22 Overall, do you think today's society is fair or unfair? [Single choice]

Completely unfair

Somewhat unfair

Can't say it's fair, but can't say it's unfair

Somewhat fair

Completely fair

Do not know

Q23 您对以下问题的信心度如何？ [矩阵量表]

Q23 How confident are you in the following statements? [Matrix scale]

|  | 完全没信心  Not confident at all | 比较没信心  Not very confident | 中立  Neutral | 比较有信心  Somewhat confident | 完全有信心  Very confident | 不确定  Uncertain |
| --- | --- | --- | --- | --- | --- | --- |
| 个人收入水平  Personal income |  |  |  |  |  |  |
| 家庭经济状况  Family economic status |  |  |  |  |  |  |
| 住房状况  Housing conditions |  |  |  |  |  |  |
| 健康状况  Health status |  |  |  |  |  |  |
| 工作状况  Job situation |  |  |  |  |  |  |
| 生活状况  Living conditions |  |  |  |  |  |  |
| 家庭关系  Family relationships |  |  |  |  |  |  |
| 人际关系  Interpersonal relationships |  |  |  |  |  |  |
| 社会地位  Social status |  |  |  |  |  |  |
| 发展机会  Development opportunities |  |  |  |  |  |  |
| 社会风气  Social atmosphere |  |  |  |  |  |  |
| 就业机会  Employment opportunities |  |  |  |  |  |  |
| 社会公平公正  Social fairness |  |  |  |  |  |  |
| 食品安全  Food safety |  |  |  |  |  |  |
| 治安状况  Public security conditions |  |  |  |  |  |  |
| 社会保障水平  Social security level |  |  |  |  |  |  |
| 医疗服务水平  Medical service level |  |  |  |  |  |  |
| 教育水平  Education level |  |  |  |  |  |  |
| 物价水平  Price level |  |  |  |  |  |  |
| 基础设施  Infrastructure |  |  |  |  |  |  |
| 环境质量  Environmental quality |  |  |  |  |  |  |

Q24 您对下列说法的同意程度是怎样的？ [矩阵量表]

Q24 What is your level of agreement with the following statements? [Matrix scale]

|  | 完全不同意  Completely disagree | 比较不同意  Somewhat disagree | 有点不同意  Slightly disagree | 有点同意  Slightly agree | 比较同意  Somewhat agree | 完全同意  Completely agree |
| --- | --- | --- | --- | --- | --- | --- |
| 未来生活将如今天一样正常运转  The future society will be functioning normally as well as today |  |  |  |  |  |  |
| 人们有能力解决未来的问题  Our society is capable of addressing future social issues |  |  |  |  |  |  |
| 人们未来的安全和保障是有保证的  Our society is capable of addressing future social issues |  |  |  |  |  |  |
| 我们会有光明的未来  Our society has a bright future |  |  |  |  |  |  |
| 一切都在失去控制  Current affairs seem to be increasingly out of control |  |  |  |  |  |  |
| 人们的安全是有保障的  We live in a secure and reliable era |  |  |  |  |  |  |
